# Evaluation of selected categories of pet treats using in vitro assay and texture analysis

**DOI:** 10.1093/tas/txaa064

**Published:** 2020-05-21

**Authors:** Fei He, Grace Holben, Maria R C de Godoy

**Affiliations:** Department of Animal Sciences, University of Illinois, Urbana, IL

**Keywords:** digestibility, gastrointestinal obstruction, in vitro assay, pet treat, safety, texture

## Abstract

Treats are important contributors to the economics of the U.S. pet product industry. Not only do pet owners use them to build an emotional bond or interact with their pets, but treats also can deliver functional or health benefits. The objective of this study was to evaluate the digestion and safety of selected commercial treats by measuring their in vitro dry matter disappearance (DMD) using the modified in vitro method of Boisen and Eggum, which was developed to simulate in vivo digestibility of nonruminant animals. Twenty-five commercial treats were classified into six categories based on their appearance, size, and functionality. These categories included biscuit, chew, dental, meat product, rawhide, and cat treat. Each commercial product was analyzed in triplicate and in vitro DMD was calculated after enzymatic digestion and incubation. A wide variation in DMD was observed among and within different treat categories in both gastric and gastric + small intestinal phases of digestion. For the gastric phase, DMD ranged from 8.40% to 92.20%, whereas intestinal phase digestion had a DMD range of 35.10–100% (*P* < 0.05). In general, treats from meat products, dental, chew, biscuit, and cat treat categories had a high DMD (>85%) after the intestinal phase, whereas DMD of rawhide treats varied from 35.10% to 95.70%. Principal component analysis, in addition, has visually shown that rawhide treats displayed the largest portion of variation from the other treats. A low DMD at gastric phase is a concern because it may pose a risk for gastrointestinal blockage and intolerance, particularly for treats of large size that remained intact during this phase. In vitro DMD results can be used as a potential predictor of in vivo digestibility, facilitate recommendations about pet treat safety for professionals and manufacturers in the pet industry, and assist pet owners in the treat selection process and with treat purchasing decisions.

## INTRODUCTION

The total U.S. retail sales of pet treats has reached over $6 billion in 2017 as estimated by Packed Fact (Sprinkle, 2017). Treats for dogs account for 85% of the market share against a 15% for cat treats. Among the different categories of dog treats, indulgent treats (i.e., meat products) are the largest product segment, at 35% of the overall pet treat market, followed by rawhide/long-lasting edible chews, dental chews/treats, and functional treats other than dental (Sprinkle, 2017). As the relationship between companion animals and their pet parents continue to evolve, pet treats are used for multiple reasons, which is represented by the wide diversity and segmentation of this market. Pet treats are often used as a demonstration of affection but can also serve as positive reinforcement during training and to deliver functional nutrients intended to manage specific conditions or to enhance pet wellness as it is the case of dental treats.

With the increase in the popularity and consumption of pet treats, pet parents have spent closer attention to the safety of these products. Bones are the most common esophageal foreign bodies that have been reported in dogs (Luthi and Neiger, 1998). In addition, bone category has been reported as the least digestible treat category in a previous study (de Godoy et al., 2014). Chew treats, rawhide, and some dental chew treats have also been reported to cause severe obstruction within the esophagus of dogs (Leib and Sartor, 2008). Therefore, understanding the digestibility of commercial treats is important to determine their safety for consumption. Safe treats must be partially or completely digested in the gastric and intestinal phases to prevent oral and gastrointestinal perforations or blockage (de Godoy et al., 2014). Currently, there’s limited studies on the digestibility of various commercial pet treats (Hooda et al., 2012; de Godoy et al., 2014).

There is, therefore, a need in the pet food field for a noninvasive method to evaluate treat products and estimate their safety and overall digestibility upon consumption. An effective method to test for these characteristics without risking the animal’s well-being and health is through in vitro assay technique. The test mimics the pet’s digestive processes by simulating the gastric and small intestinal conditions. The objective of the present study was to measure in vitro dry matter disappearance (DMD) characteristics of selected categories of commercially available pet treats and evaluate the relationship and potentiality of using texture profiles as a predictor of in vitro digestibility in order to bridge the lack of knowledge between texture profiles, safety, and digestibility of commercially available treats.

## MATERIALS AND METHODS

No animal experimentation was utilized in this study and for this reason there was no need for animal protocol approval by the Institutional Animal Care and Use Committee at the University of Illinois at Urbana-Champaign

### Sample Categories

A total of 25 commercial pet treats were investigated in this study. Treats were divided into six categories based on their size, appearance, and functionality. These categories include: meat product (MP), dental (DE), chew (CH), rawhide/rawhide like (RH), biscuit (BI), and cat treat (CT). All of the treats tested were commercially available and purchased at local pet stores or online. The packages were kept closed until in vitro analysis was initiated to avoid changes in the original texture or physico-chemical properties of these products. Samples were selected randomly from different manufactures and aimed to represent a wide variety of current products in the pet food market in the United States.

The meat product was comprised of seven treats: chicken and beef sausage, duck jerky, chicken meatball, dehydrated haddock fillet, bacon/flour mix, and chicken/flour mix. Dental category (six treats) consisted of edible bone with no wheat gluten (for dogs 7–11 kg and 11–23 kg, respectively), edible twist design bone, edible bone with wheat gluten, vegetable-based alligator-shape treat with no gluten, and twisted mini-size bones. Rawhide/rawhide-like category included four treats: beef hide bones, rawhide bone, beef hide stick with wheat gluten, and rawhide-like twisted pig skin. Chew category consisted of three products, including edible bones with wheat gluten and rawhide-free bone-contained vegetable and chicken mixture; whereas the biscuit category comprised two bone-shaped products with no wheat gluten at various sizes (i.e., mini and large). In addition to dog treats, we have also included three cat treats, including string-shaped treat, extruded-filled chicken flavor kibble, and a protein-rich bite size treat. Individual description and dimensions of treats evaluated in this study are presented in Table 1.

**Table 1.** Treat categories, main ingredient description, and product dimensions of select pet commercial pet treats tested in vitro

Treat categories	Treat description	Treat dimension L × W × H (mm)
Meat product		
Meat product 1 (MP1)	Chicken, soy grits, beef, and soy flour	51 × 30 × 11
Meat product 2 (MP2)	Duck, turkey, soy flour	70 × 17 × 6
Meat product 3 (MP3)	Chicken, soy grits, beef, and soy flour	27 × 25 × 24
Meat product 4 (MP4)	Dehydrated haddock	42 × 31 × 4
Meat product 5 (MP5)	Beef and bacon with wheat flour	40 × 22 × 6
Meat product 6 (MP6)	Chicken and soybean meal with wheat flour	70 × 15 × 7
Meat product 7 (MP7)	Freeze dried lamb liver	24 × 17 × 12
Dental		
Dental 1 (DE1)	Edible bone—no wheat gluten	71 × 20 × 16
Dental 2 (DE2)	Edible bone—no wheat gluten	80 × 24 × 20
Dental 3 (DE3)	Edible bone—no wheat gluten	116 × 30 × 16
Dental 4 (DE4)	Edible bone—with wheat gluten	102 × 17 × 12
Dental 5 (DE5)	Alligator shape	70 × 31 × 23
Dental 6 (DE6)	Twisted bone shape	87 × 25 × 23
Rawhide and rawhide like		
Rawhide 1 (RH1)	Chicken breast and beef hide	91 × 17 × 15
Rawhide 2 (RH2)	Bone-shaped beef hide	98 × 30 × 30
Rawhide 3 (RH3)	Twisted pig skin—no rawhide	128 × 19 × 18
Rawhide 4 (RH4)	Stick-shaped beef hide with wheat gluten	138 × 22 × 22
Chew		
Chew 1 (CH1)	Edible bone—with wheat gluten	31 × 24 × 14
Chew 2 (CH2)	Edible bone—with wheat gluten	119 × 35 × 24
Chew 3 (CH3)	Rawhide-free, vegetable and chicken mixture	58 × 26 × 19
Biscuit		
Biscuit 1 (BI1)	Edible bone-shaped—no wheat gluten	97 × 38 × 19
Biscuit 2 (BI2)	Edible bone-shaped—no wheat gluten	22 × 10 × 9
Cat treat		
Cat treat 1 (CT1)	Edible string shape with wheat gluten	215 × 4 × 4
Cat treat 2 (CT2)	Extruded-filled chicken flavor kibble	11 × 9 × 6
Cat treat 3 (CT3)	Semimoist protein-rich bite size	11 × 11 × 5

### In Vitro Digestibility Experiment: A Multienzyme Incubation and Filtration Method

In vitro DM digestibility was analyzed using the modified method of Boisen and Eggum, which was developed to be similar to in vivo digestibility result of nonruminant (Boisen and Eggum, 2005). This modified method was published from our previous work (de Godoy et al., 2014) and was suitable for various samples based on pet treat shape and sizes. Specifically, a mixture of phosphate buffer (0.1 M, pH 6.0) and HCl-pepsin solution was added to the container with whole treat. The pH of this mixed solution was adjusted to pH 2.0. After the addition of 5 mL of chloramphenicol solution, containers were sealed and incubated at 39 °C for 6 h to mimic gastric digestibility. Then triplicates of gastric stage were filtered through polyester fabric, rinsed, and dried at 57 °C.

Following gastric phase, pH was adjusted to 6.8 by the addition of sodium hydroxide solution (0.5 M). Small intestinal digestibility was estimated after further addition of pancreatin-phosphate buffer mixture (0.2 M, pH 6.8). After 18 h of incubation at 39 °C, triplicates of intestinal stage were filtered through polyester fabric, rinsed, and dried at 57 °C. Recovered residues from both gastric and intestinal stages were used to determine DMD as below:

$$\begin{array}{l} DMD=\left(1-DM\;residue/DM\;initial\right)\;\times \;100\%. \end{array}$$

Visual representation for the undigested materials was provided by photographs (Supplementary Fig. 1).

### Texture Profile Analysis

The Bourne analysis (Bourne et al., 1978) was used to determine break strength and resistance to penetration (hardness) in selected treat samples. Texture profile analysis was conducted using a texture analyzer (TA.HD Plus; Texture Technologies Corp., Scarsdale, NY/Stable Microsystems, Godalming, UK) equipped with heavy duty platform (HDP/90) with blank plate. The TA settings of instrument included 2.0 mm/s pretest speed, 1.0 mm/s test speed, and 10 mm/s posttest speed, and load cell capacity was 30 kg. Samples were removed from package just prior to testing to avoid moisture loss or uptake from the atmosphere, and each sample was analyzed in triplicate at room temperature (20 °C). A force–time curve was plotted and peak force for the compression was designated to calculate hardness. Data on the force applied was deduced to determine the hardness and break strength using accessories from the same manufacturer as listed below.

Break strength of six selected treats were measured using a three-point bend rig (A/3PB) accessory (Texture Technologies Corp., Scarsdale, NY/Stable Microsystems, Godalming, UK) attached to the texture analyzer. These treat samples include a meat product (MP6), two dental treats (DE4 and DE6), two rawhide treats (RH1 and RH2), and one biscuit treat (BI1). These samples were chosen because of their size and shape. Each treat sample was analyzed in triplicate. Starting 3 cm above the sample, the crossbar descended and exerted sufficient force necessary to fracture the sample. This force (kg) was recorded, measurements were averaged in replicate, and values were reported as mean maximum force.

Resistance to penetration (hardness) was measured using a 2-mm probe (P/2) accessory (Texture Technologies Corp., Scarsdale, NY/Stable Microsystems, Godalming, UK) attached to the texture analyzer. This probe was used to mimic the biting and chewing behavior of pets. The probe approaches the sample and once a 5-g force is attained, a rapid rise in force is observed. The probe returns to its original starting position when a penetration distance of 3 mm from the trigger point is reached. The mean penetration energy (area under the curve) is measured as an indication of the hardness. Out of the 25 samples tested in this in vitro study, only 22 samples (i.e., 7 MP, 6 DE, 4 RH, 3 CH, and 2 BI treats) could be evaluated for hardness as the three cat treats were too small to be adequately tested by the texture analyzer. Each treat was analyzed in duplicate, maximum force data for each treat was recorded, averaged, and reported as mean maximum force (1 gram-force = 0.00981 N). Within the 22 samples analyzed, 7 samples exceeded the maximum detectable force of 30,000 g of the texture analyzer as denoted on (Table 2).

**Table 2.** Texture profile analysis of selected commercial treats

Treat categories	Break strength; mean max. force (kg)	Hardness; mean max. force (kg)
Meat product		
Meat product 1 (MP1)	NA	0.6 ± 0.12
Meat product 2 (MP2)	NA	0.7 ± 0.09
Meat product 3 (MP3)	NA	0.4 ± 0.12
Meat product 4 (MP4)	NA	3.7 ± 0.53
Meat product 5 (MP5)	NA	2.1 ± 0.30
Meat product 6 (MP6)	0.3 ± 0.02	1.1 ± 0.068
Meat product 7 (MP7)	NA	1.5 ± 0.17
Dental		
Dental 1 (DE1)	NA	12.5 ± 0.30
Dental 2 (DE2)	NA	19.0 ± 1.09
Dental 3 (DE3)	NA	8.8 ± 0.37
Dental 4 (DE4)	5.7 ± 0.76	25.2 ± 1.45
Dental 5 (DE5)	NA	30 (overload)
Dental 6 (DE6)	NA	30 (overload)
Rawhide and rawhide like		
Rawhide 1 (RH1)	11.1 ± 0.30	30 (overload)
Rawhide 2 (RH2)	NA	30 (overload)
Rawhide 3 (RH3)	NA	30 (overload)
Rawhide 4 (RH4)	NA	30 (overload)
Chew		
Chew 1 (CH1)	NA	10.0 ± 0.77
Chew 2 (CH2)	NA	30 (overload)
Chew 3 (CH3)	NA	22.7 ± 3.56
Biscuit		
Biscuit 1 (BI1)	12.2 ± 0.25	8.9 ± 3.1
Biscuit 2 (BI2)	NA	3.9 ± 0.55
Cat treat		
Cat treat 1 (CT1)	NA	NA
Cat treat 2 (CT2)	NA	NA
Cat treat 3 (CT3)	NA	NA

NA represents samples not able to be tested due to size or shape.

### Statistical Analysis

Data were analyzed with the MIXED procedure (SAS Inst. Inc., Cary, NC). The statistical model included the fixed effect of treat category, treat type, and digestibility phases. Results of least-square means were compared between treatments, and Tukey adjustment was used to control experiment error. Statistically significance was defined by a probability of *P* ≤ 0.05. Principal components analysis (PCA) and scattered plot were developed using Origin Pro 8 (Origin Lab Inc.) in order to allow an easier visualization of the acquired data.

## RESULTS AND DISCUSSION

### In Vitro DM Digestibility

**Figure 1.**
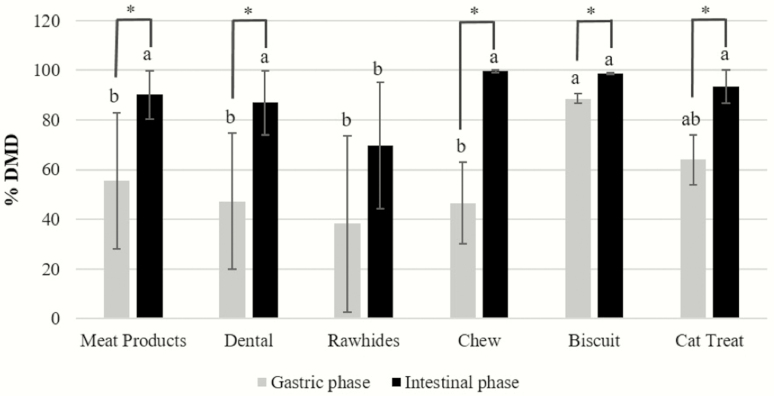
Average in vitro DMD by category of pet treats. *Average mean treat category effect between in vitro phases (*P* < 0.05). ^a,b^Average mean values for treat category within in vitro phase not sharing common superscript letters differ (*P* < 0.05).

The tested samples had a wide range of in vitro DMD, ranging from 8.41% to 92.15% in gastric phase and 35.14% to 99.98% in intestinal phase (*P* < 0.05; Table 3). Meat products were highly variable in DMD in gastric phase, varying from 16.60% to 92.15%. While in intestinal phase, DMD ranged from 72.45% to 99.37% (Table 3). Treats in all categories, except rawhides, had a greater (*P* < 0.05) DMD after 18 h of in vitro incubation when compared with the gastric phase (Fig. 1).

**Table 3.** In vitro gastric and intestinal DMD of pet treats

Treat categories	Gastric phase (6 h)	Intestinal phase (18 h)
Meat product		
Meat product 1 (MP1)	49.88^b^	87.71^c^
Meat product 2 (MP2)	65.63^ab^	85.25^c^
Meat product 3 (MP3)	92.15^a^	99.37^a^
Meat product 4 (MP4)	86.13^a^	99.17^ab^
Meat product 5 (MP5)	38.22^b^	97.81^ab^
Meat product 6 (MP6)	16.60^bc^	72.45^d^
Meat product 7 (MP7)	39.55^b^	88.87^bc^
Dental		
Dental 1 (DE1)	86.24^a^	94.66^ab^
Dental 2 (DE2)	38.27^d^	89.62^bc^
Dental 3 (DE3)	73.39^b^	98.97^a^
Dental 4 (DE4)	42.93^c^	88.85^bc^
Dental 5 (DE5)	14.62^f^	62.01^d^
Dental 6 (DE6)	27.82^e^	87.07^c^
Rawhide and rawhide like		
Rawhide 1 (RH1)	15.63^c^	70.73^bc^
Rawhide 2 (RH2)	42.05^b^	76.98^ab^
Rawhide 3 (RH3)	8.41^c^	35.14^d^
Rawhide 4 (RH4)	86.9^a^	95.74^a^
Chew		
Chew 1 (CH1)	56.35^a^	99.01^b^
Chew 2 (CH2)	55.58^a^	99.81^a^
Chew 3 (CH3)	27.51^b^	99.98^a^
Biscuit		
Biscuit 1 (BI1)	87.21	98.41
Biscuit 2 (BI2)	89.40	98.90
Cat treat		
Cat treat 1 (CT1)	71.96^a^	99.82
Cat treat 2 (CT2)	52.65^c^	94.31
Cat treat 3 (CT3)	67.08^b^	86.33

^a–f^Means without a common superscript letter within a column and treat category differ (*P* < 0.05).

Dry matter disappearance of DE treats were highly variable in gastric phase, varying from 14.62% to 86.24% but were highly digestible at intestinal phase (>87%), except for DE5 that had a DMD of 62.01% (Table 3). These findings are in accordance with our previous study (de Godoy et al., 2014), which indicated that the use of wheat gluten may not lead to lower DMD because the variation in treat recipes and processing may result in different interactions among ingredients and in final product digestibility.

Rawhide is one of the conventional edible dog chews. A typical rawhide production process begins with rendering or tanning animal hides, which are then cut and trimmed into desired sizes. A final product often consists of multiple layers of hides (Devahastin, 2010). Rawhide strips knotted on the ends to resemble bones, for example, may provide abrasion for cleaning teeth by removing tartar and massaging the gums, which is not provided by the typical canine dog food (Axelrod, 2006). However, the indigestible leather fragments of rawhide chews swallowed by the dogs may lead to gastrointestinal blockage or diarrhea (Simone et al, 1994). In our study, this category contained rawhide and rawhide-like products, which had a wide range of gastric DMD values, with RH3 being the least digestible (8.41%), followed by RH1 (15.63%), RH4 being the most digestible (86.90%) and RH2 showing intermediate DMD (42.05%; Table 3). No statistical differences in DMD were observed between RH1 and RH3 at gastric phase in contrast to their intestinal phase digestibility. Compared to RH category, CH treats were all highly digestible at intestinal phase (>99%), CH1 and CH2 were very similar in DMD at gastric phase (>55%), while CH3 had a lower (*P* < 0.05) DMD of 27.51% in gastric phase compared to the other two treats. de Godoy et al. (2014) also reported similar DMD ranges for rawhide and chew treats during gastric (14–73% and 28–85%, respectively) and intestinal phases of 76–100% and 55–100%, respectively.

Baked treats are one of the most popular treats and often available as hard biscuit products. The biscuit treats are, commonly, produced by mixing grain flour and water to produce a dough. The dough is then shaped by rotary molding and stamping and baked in a cool oven to make hard and crunchy products (Devahastin, 2010). Glazed-coated treat may also be produced as value-added product during pet treat production (Mair, 2003). The BI treats analyzed herein were highly disintegrated after gastric phase and showed a high gastric and intestinal DMD of 87.21–89.40% and 98.41–98.90%, respectively. These results indicated that these treats would not pose a risk of gastric blockage when consumed in whole by pets. The two BI treat samples were produced from the same company but in two different sizes (large vs. mini); their similar DMD in both gastric and intestinal phases also confirmed that our in vitro method was not affected by the size of treat as it was expected. In addition to dog treats, three CT were also tested in this study, and all of them had a moderate to high gastric DMD from 52.65% to 71.96% and high intestinal digestibility (>86%; Table 3).

### PCA Analysis

**Figure 2.**
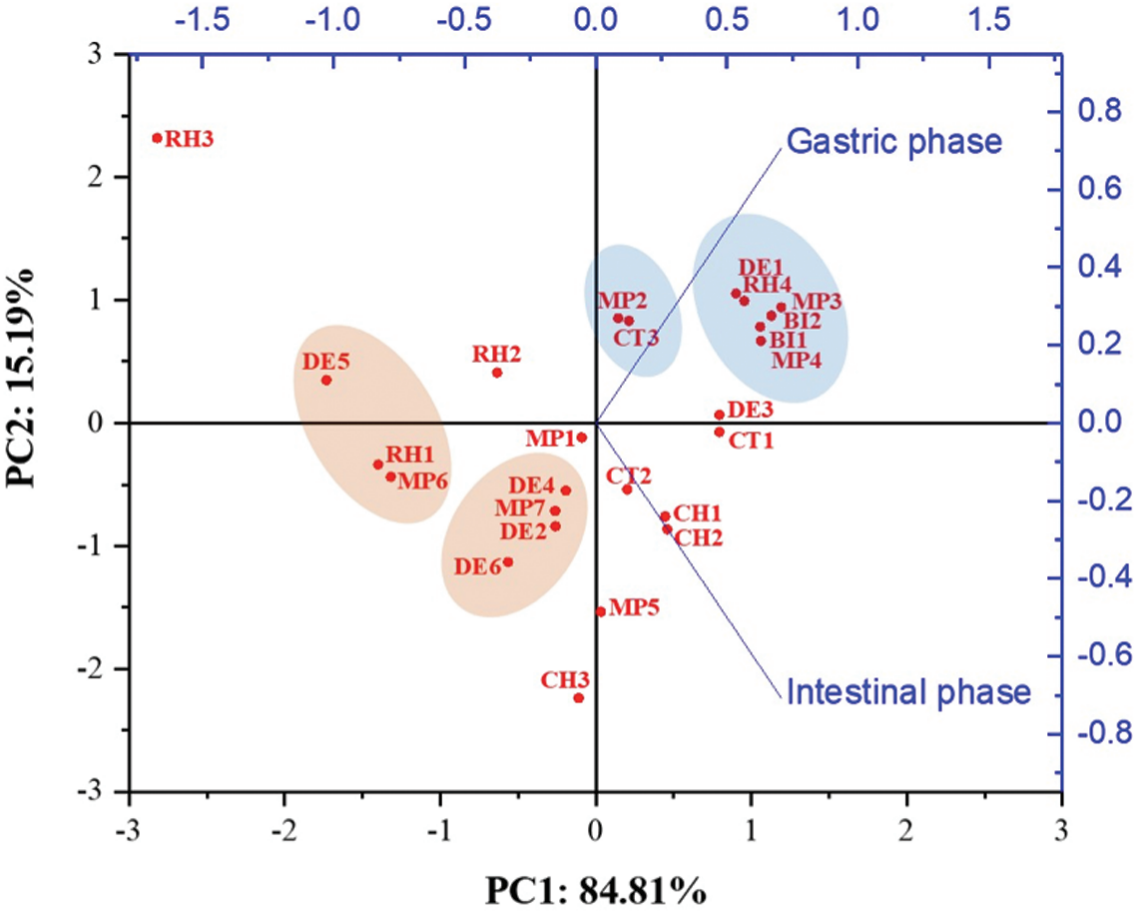
PCA of in vitro gastric and intestinal DMD of commercial treats.

As is shown in PCA 2D biplot analysis (Fig. 2), the two variables (gastric and intestinal phases) were labeled as vectors, and each sample was labeled in points as observations. Meat product 2 and CT3 showed similar digestibility in both gastric and intestinal phases. Meat product 3 and MP4, DE1, RH4, and BI1 and BI2 samples were all highly digestible, and all of them had similar DMD in both gastric and intestinal phases as depicted by their clustering in the PCA plot. The high DMD suggests potentially good utilization of the treats by the dogs. On the contrary, MP6, RH1, and DE5 exhibited low gastric DMD (14.62–16.60%) and moderate DMD in intestinal phase (62.01–72.45%). A moderate gastric DMD was observed for treats MP7, DE2, DE4, and DE6, which resulted in their clustering in the PCA plot. Rawhide 3 was the furthest away from the model, indicating that this treat sample displayed the largest portion of unexplained variation and also had the lowest DMD in gastric phase (8.41%) and intestinal phase (35.14%) among all samples. The low DMD poses a concern for dogs that tend to consume large pieces without much mastication prior to swallowing and could pose a risk for gastric blockage or gastrointestinal intolerance, especially because the treat matrix stayed mostly intact in one piece as indicated by the photographs (Supplementary Fig. 1).

### Texture Profile Analysis

To our knowledge, this is the first study to report in vitro DMD versus texture profiles in dog treats. Hardness (maximum force to maintain extrusion; g) provides an indication of the force required to press the food during mastication. Break strength, in this case, indicated how flexible or malleable the sample was. In this study, we used a 2-mm probe to mimic the animal tooth in the biting behavior and a three-point bend rig to perform the break strength testing. All meat products showed the lowest hardness (all below 5 kg) compared to other categories, including DE, RH, CH, and BI (Table 2), despite the wide variation observed in the DMD of MP (16.60 % to 92.15%) in gastric phase. MP6 had a low gastric phase DMD and also showed low hardness and low break strength, suggesting that this sample would be easy to bite or break upon consumption. It is worth noting that hardness of treats can be affected by factors including viscosity, processing method, and macronutrient composition (e.g., fat, protein, or carbohydrate; Hooda et al., 2012). Biscuit category exhibited low to medium hardness (between 5 kg and 10 kg), as well as break strength, and this category had high in vitro DMD of over 87% in gastric phase.

Dental, RH, and CH categories showed high variations between treat types, DMD, and texture profiles. Among these three categories, we observed that samples with a high gastric DMD did not always show low hardness, but samples with low DMD usually had high hardness. For example, RH4 had a high DMD and hardness. Dental 5, DE6, RH1, and RH3 had low DMD and high hardness. In fact, these samples overloaded the texture analyzer, exceeding the load cell capacity of 30,000 g. For these samples, we assumed that minimum hardness would be equal 30,000 g, but it could be greater than that. Samples with medium DMD (40–50 %) in these categories, DE4 and RH2 in this case, exhibited medium or high hardness, respectively.

**Figure 3.**
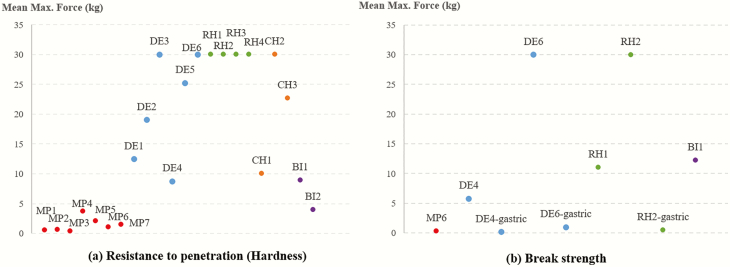
Texture profile (hardness and break strength) analysis in selected dog treats. Samples with overloading message from the instrument were shown with 30 mean max. force (kg), indicating that they were over the range of instrument capacity.

We also observed that samples with similar DMD may pose different texture profiles. For example, CH1 and CH2 were not significantly different in their gastric or intestinal DMD (Table 3); however, their resistance to penetration (hardness) were quite different (Fig. 3a). Chew treat 1 showed low to medium hardness, while CH2 overloaded the texture analyzer.

**Figure 4.**
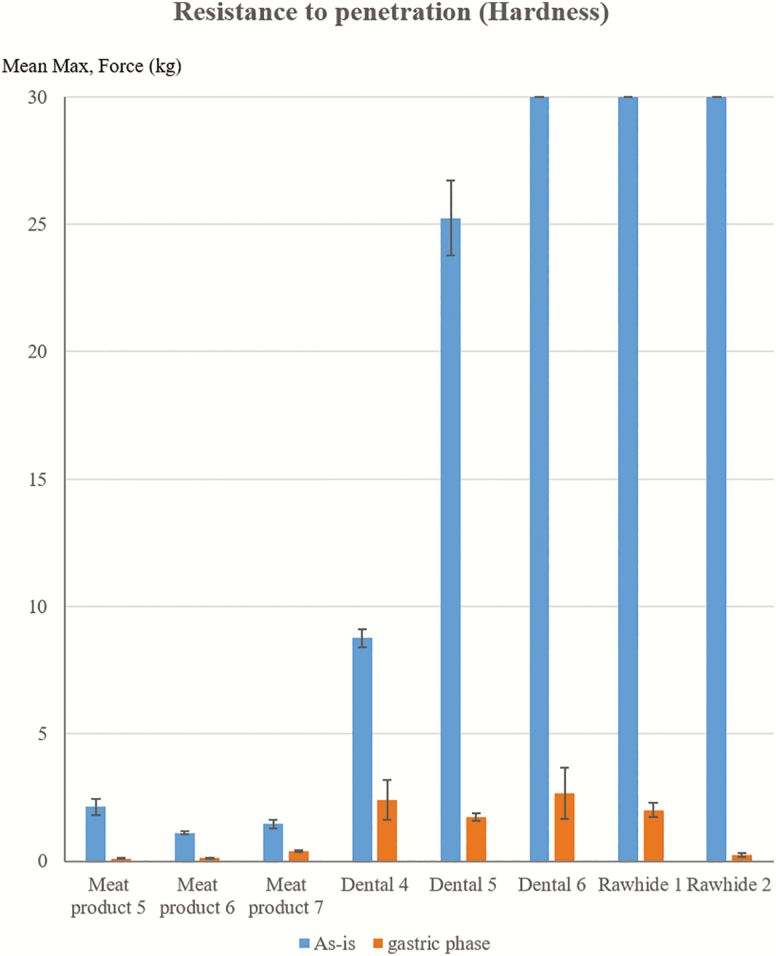
Comparison of texture profile (hardness) of selected dog treats between as-is and gastric phase. Samples with overloading message from the instrument were shown with 30 mean max. force (kg), indicating that they were over the range of instrument capacity.

Since samples with low DMD are of most concern as they may pose a risk for gastric or intestinal blockage or perforation (de Godoy et al., 2014), the gastric residues from selected samples with low DMD were also tested for texture profiles (Fig. 4) and a three-point rig for break strength (Fig. 3b). The hardness of gastric phase residues was significantly lower compared to as-is form in all three categories tested (i.e., MP, DE, and RH).

## CONCLUSIONS AND IMPLICATIONS

In conclusion, multienzyme incubations in two steps followed by a collection of undigested materials by filtration have been developed and tested in commercial treats for general and routine use in practical evaluation of pet treat digestibility. This method has been demonstrated to be able to predict gastric and intestinal digestibility and safety of pet treats. Texture profiles were, for the first time, reported in accordance with in vitro DMD, especially to evaluate samples with low DMD. The low DMD may pose a concern for dogs that tend to consume large pieces without much mastication prior to swallowing and could pose a risk for gastric blockage or gastrointestinal intolerance. Thus, in this in vitro study, treats that stayed mostly intact in one piece (as indicated by the pre- and post-in vitro assay treat images) could pose a risk upon consumption for the pet animal. However, texture analysis of both as-is sample and the gastric residues suggested that the malleable characteristics of these treats, while in solution, would most likely not pose a risk for gastric or intestinal perforation. Further texture analysis is still needed (such as measurements of fracturability, adhesiveness, springiness, cohesiveness, chewiness, and resilience) in order build a correlation model in accordance with in vitro DMD for the purpose of developing additional predictors of treats characteristics related to their safety for pet animals.

The information from this study may provide guidance in pet treats formulation and processing. This information can also be leveraged to assist pet owners about adequacy and safety of treats commonly purchased. For treat manufacturers, it is important to verify that the treats are digestible and safe for consumption. Variation in treat recipes and processing methods may lead to differences in the texture and digestibility of the final product. Additional in vivo evaluation of treats is still needed once a product is suggested as safe using in vitro DMD assay in order to verify nutritional or functional properties of the product on targeted species.

## SUPPLEMENTARY DATA

Supplementary data are available at *Translational Animal Science* online.

**Supplementary figure 1.** Pictorial representation of *in vitro* digestion of selected pet treat categories

txaa064_suppl_Supplementary_Figure_1
