# Supplementary figures and images for: Evaluation of selected categories of pet treats using in vitro assay and texture analysis
Source: Transl Anim Sci. 2020 May 21;4(2):1023–30. doi: 10.1093/tas/txaa064 (PMC7332238; doi:10.1093/tas/txaa064)

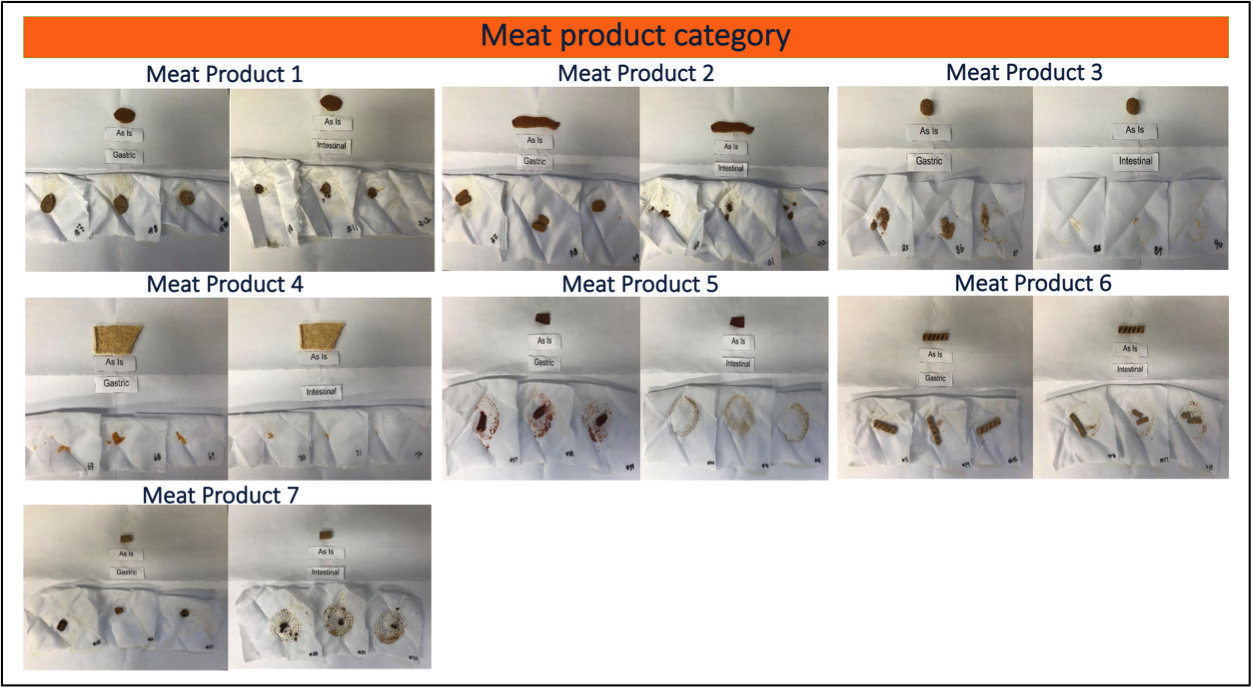

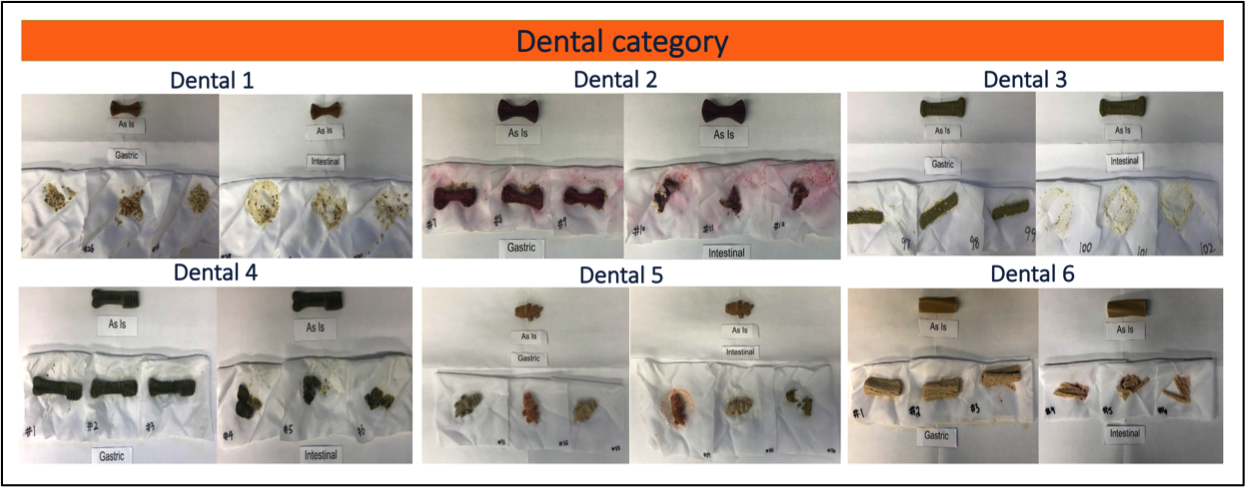

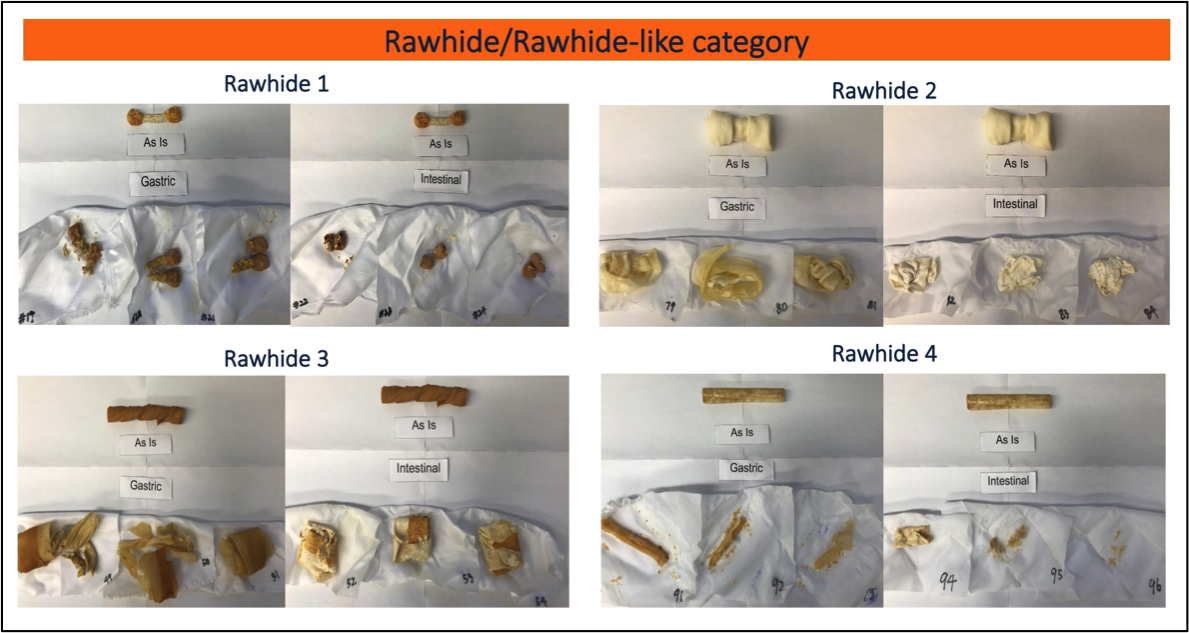

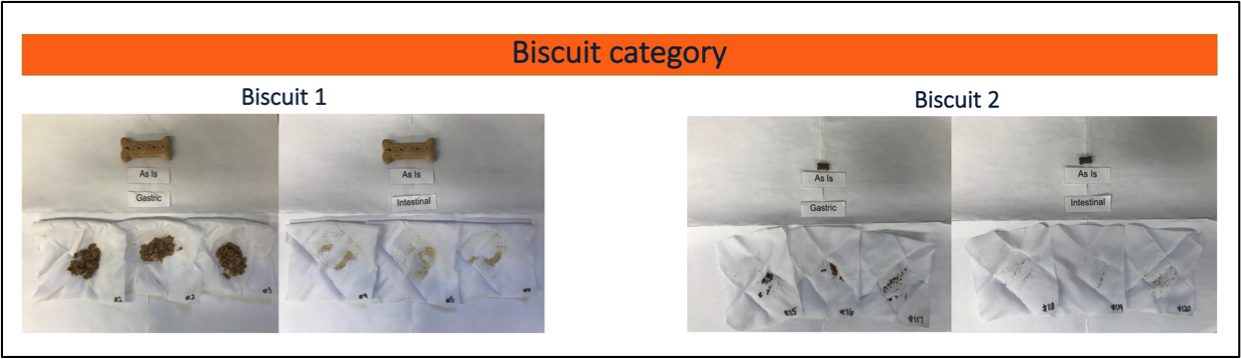

Supplement: txaa064_suppl_Supplementary_Figure_1 [file txaa064_suppl_supplementary_figure_1.docx]
